# An Unusual Case of Situs Inversus in a Premature Neonate

**DOI:** 10.7759/cureus.102830

**Published:** 2026-02-02

**Authors:** Natalie Elazar, Marissa G Castronovo, Edith M Spencer-Morales

**Affiliations:** 1 Osteopathic Medicine, Nova Southeastern University Dr. Kiran C. Patel College of Osteopathic Medicine, Davie, USA; 2 Neonatology, Wellington Regional Hospital, Wellington, USA

**Keywords:** ciliary dyskinesia, dextrocardia -, kartagener's syndrome, preterm neonate, primary ciliary dyskinesia (pcd), rare congenital anomaly, situs inversus, situs inversus with dextrocardia

## Abstract

Situs inversus, also known as a mirror image, is the transposition of all major organs in the body from left to right. Situs inversus totalis is a rare condition characterized by the complete inversion and mirroring of organs, including the cardia, lungs, and all abdominal contents. It follows an autosomal recessive pattern of inheritance, with an incidence of approximately one in 8,000 live births. Early prognosis is important due to the risk of complications associated with this syndrome. In this case report, we will examine a premature infant with isolated situs inversus, which was discovered postnatally.

A male neonate was delivered via vaginal delivery at 33 weeks and three days of gestation to a 32-year-old G1P1 mother. Membranes were ruptured for 22 days, the amniotic fluid was clear, and the presentation was compound. Birth weight was 2.385 kg, with Apgar scores of 6 and 7 at one and five minutes, respectively. Treatment at delivery included positive pressure ventilation (PPV) via mask, positive end expiratory pressure (PEEP) (neopuff), and gastric suction. The patient was admitted immediately to a level 3 neonatal intensive care unit (NICU) following delivery for preterm respiratory distress. Chest X-ray chest/abdomen showed mild hyperinflation of both lungs, with perihilar streakiness present. Dextrocardia with situs inversus totalis was noted. There was no pleural effusion or pneumothorax. There was mild dextrocurvature of the lower thoracic spine. The tip of the orogastric (OG) tube is seen to project over the mid-gastric body. The findings may represent transient tachypnea of the newborn (TTN).

This case highlights the significance of early identification of situs inversus in neonates when presenting with respiratory distress. While Kartagener's syndrome is a common condition associated with situs inversus, not every patient will exhibit the complete triad or have recognizable genetic variants associated with primary ciliary dyskinesia. In this case, genetic testing identified likely pathogenic variants in LAMA2 and NDUFAF5, neither of which is associated with laterality defects, suggesting the patient's situs inversus is likely idiopathic. Additionally, it highlights the importance of comprehensive genetic testing to rule out known causes of isolated situs inversus, identify incidental yet potentially significant findings, and inform ongoing monitoring for potential neuromuscular or metabolic disorders.

## Introduction

Situs inversus, also known as a mirror image, is the transposition of all major organs in the body from left to right. When the heart is involved, it is known as dextrocardia, a condition in which the base of the heart faces to the right instead of the left [1]. Situs inversus totalis is a rare condition with complete organ inversion and mirroring, including the cardia, lungs, and all abdominal contents. This is an autosomal recessive pattern of inheritance, with the incidence being around one in 8000 live births [2]. Dextrocardia typically correlates with other structural deformities, such as a ventricular septal defect and tetralogy of Fallot [1]. Early prognosis is important due to the risk of complications associated with this syndrome.

Kartagener syndrome (KS) is a type of primary ciliary dyskinesia (PCD). Its presentation typically includes sinusitis, bronchiectasis, and situs inversus. It is also known to have a recessive inheritance pattern [3]. Cilia are involved in many major organ functions, including those of the respiratory, gastrointestinal, and reproductive tracts. They work to clear bacteria, transport nutrients, and enhance sperm mobility. KS results from mutations in genes encoding axonemal structures, causing abnormal ciliary structure and function. This disrupts the normal activity of affected organ systems [3]. Due to the many different uses of cilia in the body, there is a wide range of clinical presentations.

Mutations in DNAI1 and DNAH5 have been linked to KS and their role in ciliary function [4]. It relates to the dynein complex in cilia, which are the most commonly associated with KS. Other X-linked genes include RPGR and OFD1. The multitude of genes associated with this syndrome adds to the variety of clinical presentations. KS occurs in roughly 50% of patients with situs inversus [3].

In this case report, we will examine a premature infant with isolated situs inversus, which was discovered postnatally.

## Case presentation

Maternal and prenatal history

The pregnancy was a planned, spontaneous conception in a healthy 32-year-old gravida 1 para 1 mother who received routine prenatal care. The mother had no past medical or surgical history. Medications taken during pregnancy were limited to prenatal vitamins and acetaminophen as needed. Maternal blood type was O positive. Routine prenatal ultrasound evaluations, including anatomy scans, were unremarkable, with no structural abnormalities identified. Prenatal infectious disease screening was unremarkable, including a nonreactive syphilis screen; negative hepatitis, HIV, chlamydia, and gonorrhea screens; and rubella immunity. Group B *Streptococcus* status was unknown. The pregnancy was complicated by preterm premature rupture of membranes (PPROM), for which the mother received two doses of antenatal corticosteroids. Labor was induced, and the intrapartum course was complicated by chorioamnionitis and a category II fetal heart tracing. Medications administered during labor and delivery included clindamycin, gentamicin, and betamethasone.

Patient information

The patient was a male neonate delivered via vaginal delivery at 33 weeks and three days of gestation to a 32-year-old G1P1 mother. Membranes had been ruptured for 22 days prior to delivery, with clear amniotic fluid noted. The fetal presentation was compound. Birth weight was 2.385 kg. Apgar scores were 6 at one minute and 7 at five minutes.

History of present illness

At delivery, delayed cord clamping was performed for 30 seconds. The neonate had a weak cry and thick secretions. Initial resuscitation included drying, stimulation, and suctioning, with thick secretions extracted from the stomach. Due to cyanosis and weak respiratory effort, positive-pressure ventilation was initiated with 30% oxygen, resulting in clinical improvement. Additional delivery room interventions included positive end-expiratory pressure via Neopuff and gastric suction. The patient was admitted directly to a level III neonatal intensive care unit for management of preterm respiratory distress.

Medications and allergies

At the time of admission, the patient was not receiving any medications and had no known drug allergies. During hospitalization, amoxicillin was initiated for urinary tract infection prophylaxis following nephrology consultation.

Clinical findings

On admission, vital signs revealed a heart rate of 174 beats per minute, respiratory rate of 24 breaths per minute, blood pressure of 71/41 mmHg, and temperature of 98.8°F. The patient was considered critically ill due to the need for respiratory support with nasal prong ventilation. Physical examination demonstrated a soft and open anterior fontanelle with otherwise normal head, eyes, ears, nose, and throat findings. Respiratory examination revealed coarse breath sounds bilaterally with moderate respiratory distress. Cardiac auscultation revealed heart sounds and an apical impulse located on the right side of the chest, consistent with dextrocardia. The abdomen was soft and nondistended with active bowel sounds, no palpable masses, and a three-vessel umbilical cord. Genitourinary examination showed a preterm male with palpable testes and a patent anus. Neurologic examination demonstrated mildly decreased tone for gestational age with reflexes appropriate for age. The spine was intact without deformities. No limb abnormalities, dysmorphic features, or syndromic characteristics were identified on serial physical examinations. The patient moved all extremities well, and the skin examination was notable for acrocyanosis.

Diagnostic assessment

**Figure 1:**
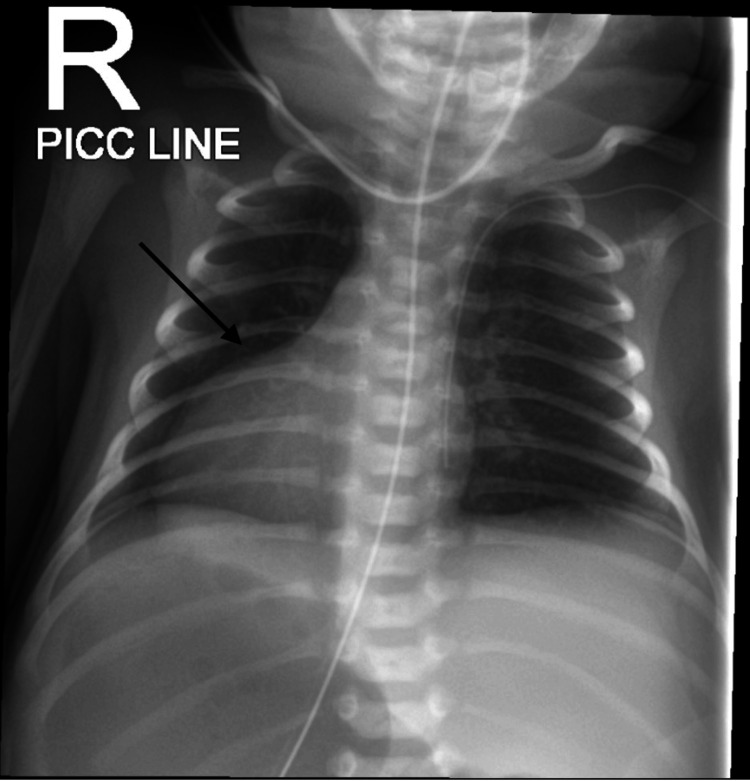
Chest X-ray Frontal Demonstrating dextrocardia.

A frontal chest radiograph demonstrated dextrocardia, with the cardiac silhouette predominantly located in the right hemithorax. A peripherally inserted central catheter and orogastric tube were visualized. There was no evidence of lung consolidation, pleural effusion, pneumothorax, or hyperinflation (Figure 1).

**Figure 2:**
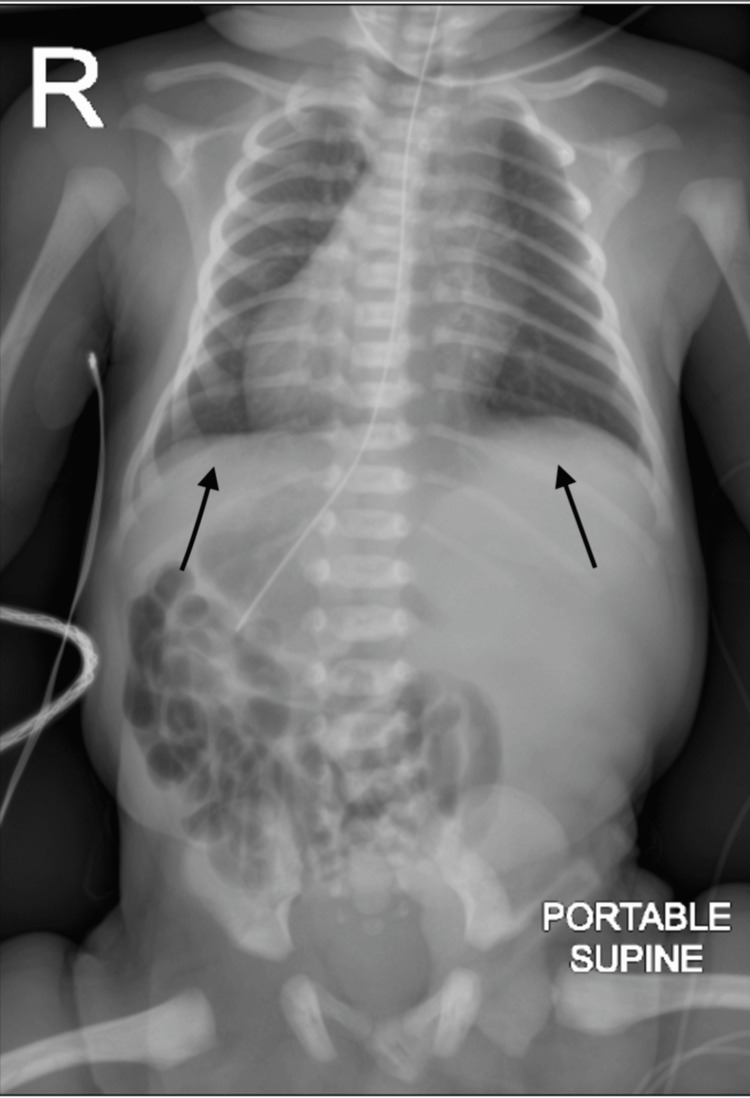
Chest X-ray Chest/Abdomen Radiograph revealing mild hyperinflation of both lungs with perihilar streakiness.

A subsequent chest and abdominal radiograph revealed mild hyperinflation of both lungs with perihilar streakiness. Mirror-image dextrocardia with situs inversus totalis was again noted, without pleural effusion or pneumothorax. Mild dextrocurvature of the lower thoracic spine was present, and the tip of the orogastric tube projected over the mid-gastric body. These findings were suggestive of transient tachypnea of the newborn (Figure 2).

**Figure 3:**
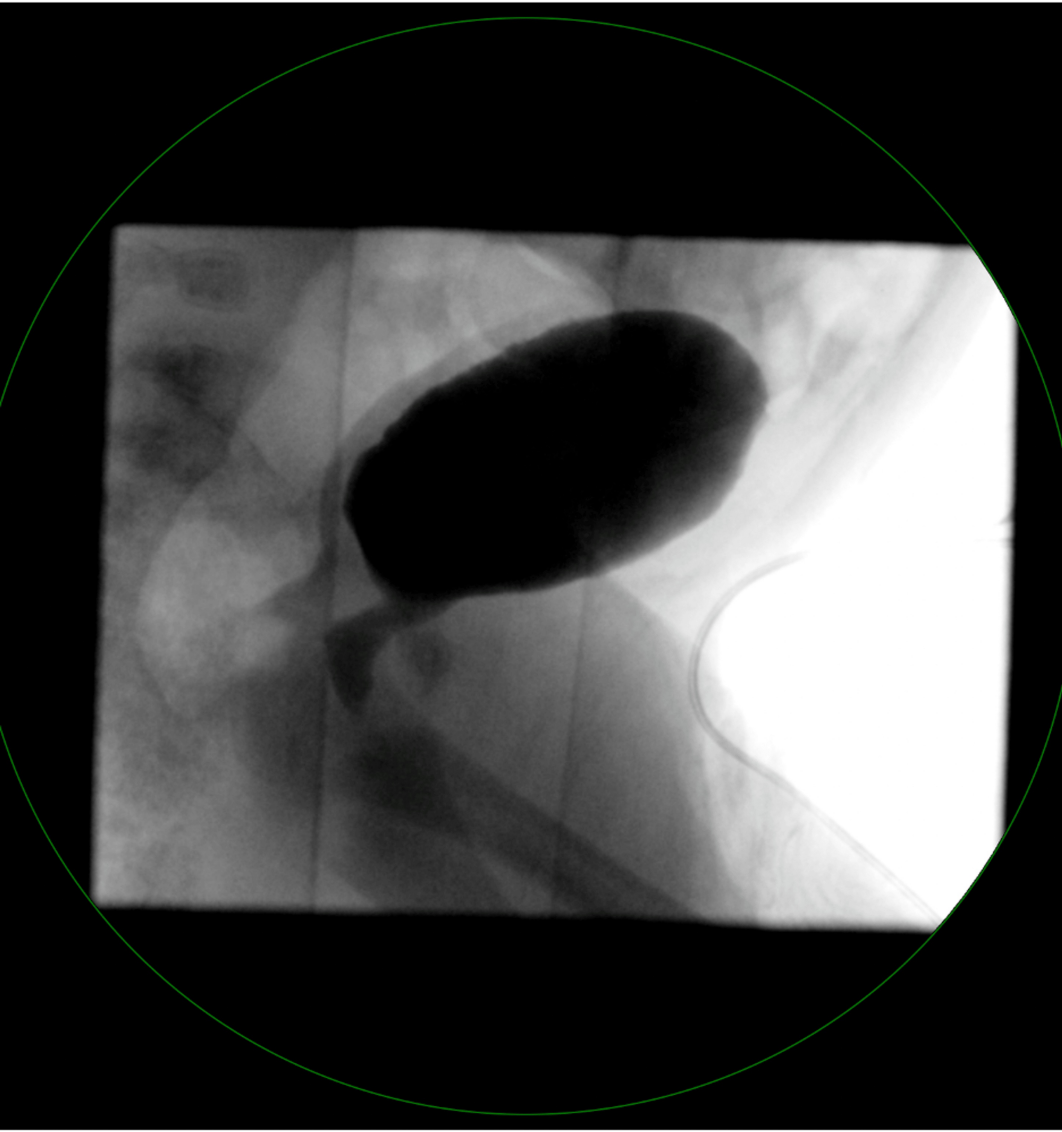
X-ray Urethrocystography Voiding Bladder urethrocystography appeared unremarkable, with no filling defects, and no vesicoureteral reflux

A voiding cystourethrogram was performed with infusion of contrast into the bladder via a Foley catheter. The bladder appeared unremarkable, with no filling defects, and no vesicoureteral reflux was observed during the examination (Figure 3). A pediatric nephrology consultation was obtained from Nicklaus Children’s Hospital (NCH), Miami, for evaluation of hydronephrosis. Based on their recommendations, prophylactic amoxicillin was initiated, with plans for continued outpatient nephrology follow-up.

Transthoracic echocardiography revealed mirror-image dextrocardia with situs inversus and atrioventricular and ventriculo-arterial concordance. A right-sided aortic arch with a normal head and neck branching pattern was identified. Intracardiac anatomy was otherwise normal, with a patent foramen ovale demonstrating left-to-right shunting. Valve structure and function were normal, as were systemic and pulmonary venous connections. The coronary artery origins were not well visualized. There was no evidence of pericardial effusion.

**Figure 4:**
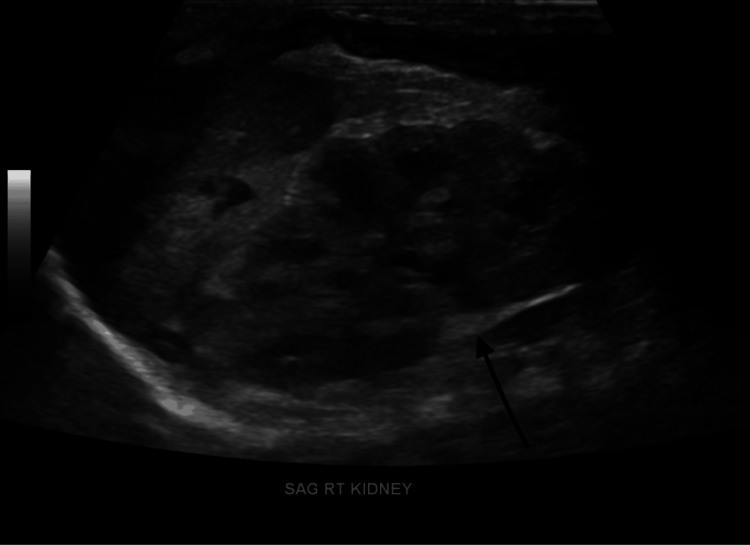
Ultrasound of the Right Kidney Ultrasound showing mild right-sided hydronephrosis.

Abdominal ultrasound demonstrated situs inversus, with the spleen identified in the right upper quadrant and appearing unremarkable. Mild right-sided hydronephrosis was noted (Figure 4).

**Figure 5:**
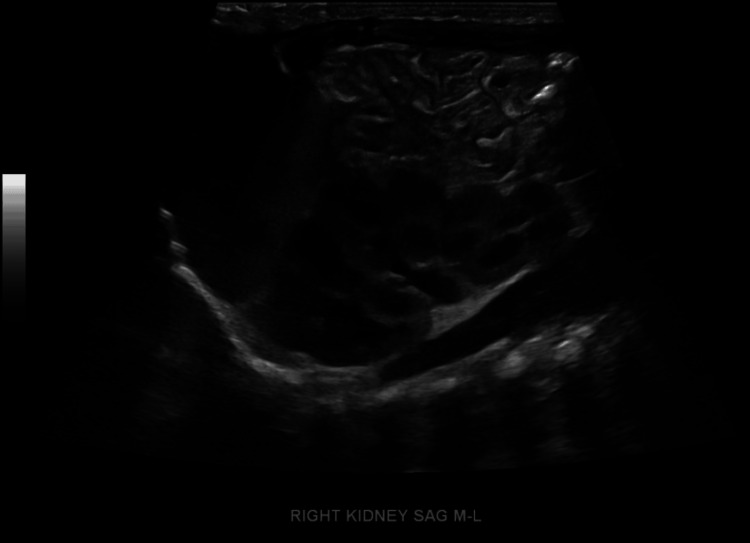
Complete Renal Ultrasound of Right Kidney Ultrasound showing mild right-sided hydronephrosis.

A subsequent dedicated renal ultrasound showed both kidneys to have normal morphology and cortical echogenicity, without stones or masses. Mild hydronephrosis of the right kidney and moderate hydronephrosis of the left kidney were observed. The left bladder jet was visualized, while the right bladder jet was not seen (Figure 5).

Genetic testing did not demonstrate findings consistent with Kartagener syndrome. Variants of probable significance were identified in LAMA2 and NDUFAF5 (Table 1). Variants in LAMA2 are associated with autosomal recessive muscular dystrophy, and variants in NDUFAF5 are linked to mitochondrial complex I deficiency. Although these variants may be pathogenic, neither gene has a known association with laterality defects or primary ciliary dyskinesia, the underlying mechanism of Kartagener syndrome. Consequently, these findings were considered incidental and did not explain the patient’s situs inversus. No pathogenic variants were identified in genes typically involved in ciliary function, and the etiology of situs inversus in this patient was therefore deemed idiopathic.

**Table 1:** Karyotype Genetic Screening Results

Gene	Variant	Zygosity
LAMA2	Likely Pathogenic Variants	Heterozygous
NDUFAF5	Likely Pathogenic Variants	Heterozygous

Differential diagnosis

The differential diagnosis included isolated situs inversus totalis, Kartagener syndrome, primary ciliary dyskinesia without the full Kartagener triad, and heterotaxy syndrome.

Therapeutic intervention

Following delivery and initial stabilization, the patient required ongoing respiratory support with nasal prong ventilation. On hospital day 3, the patient was transitioned to nasal continuous positive airway pressure, which was continued for two days. By hospital day 5, the patient was weaned to room air.

Follow-up and outcomes

Serial chest and abdominal radiographs confirmed mirror-image dextrocardia with situs inversus. Transthoracic echocardiography demonstrated normal intracardiac anatomy and normal systemic and pulmonary blood flow. Voiding cystourethrogram showed no evidence of vesicoureteral reflux. Abdominal and renal ultrasound findings were notable for hydronephrosis, predominantly affecting the right kidney. Based on specialist recommendations, urinary tract infection prophylaxis with amoxicillin was initiated, with plans for outpatient nephrology follow-up. Throughout hospitalization, the patient received routine neonatal care, tolerated enteral feeding advancement, maintained appropriate oxygen saturations, and demonstrated adequate urine and stool output. The patient was discharged home on day 24 of life with planned outpatient pediatric follow-up. Overall findings were consistent with isolated situs inversus totalis.

## Discussion

Although rare, situs inversus is compatible with a normal life expectancy; however, early diagnosis is important because common conditions may present atypically in these patients. Common conditions such as appendicitis, cholecystitis, and even heart murmurs will appear differently in these patients. While these diseases may be common, they can be life-threatening without proper treatment. 

In patient A, this condition was only discovered because a chest x-ray was needed to rule out pneumonia or transient tachypnea of the newborn. Compared to patient A, another case report has shown another instance of situs inversus in a premature neonate. Patient B in the case report was shown to have mild grunting and subcostal retractions. The lung sounds were clear and symmetrical to auscultation [2]. This infant was transferred to the neonatal intensive care unit (NICU) for 3 L of oxygen flow. While in the NICU, a chest x-ray was performed, revealing complete situs inversus. Patient B, similarly to Patient A, received a repeat chest x-ray to confirm placement of left and right markers [2]. This case report demonstrated similarities to Patient A, in which there was a premature delivery with respiratory distress and routine imaging that showed situs inversus. In both patients, it was essential to observe symptoms for recurrent bronchiectasis [2]. 

These cases also highlight the importance of distinguishing between variants of situs inversus, particularly those with and without Kartagener syndrome (KS). As noted, KS occurs in around 50% of patients with situs inversus [3]. Those that present without the syndrome are known to have specific recessive mutated genes known to cause PCD. This is likely the cause of the reduced phenotype, such as known PCD symptoms [5]. Some of these patients have identifiable genes that we can relate to their status. Gene PKD1L1, which encodes a calcium channel associated with non-motile cilia, has been distinguished. Another gene, CFAP52, is thought to play a role in cilia signaling [5]. However, the majority of situs inversus patients aren’t known to have a specific genomic explanation for their lack of PCD. Further research is needed to understand the relationship between situs inversus with and without KS. 

A published case series evaluating comorbidities associated with situs inversus reported that congenital heart defects were present in approximately 46.5% of cases, while renal disorders accounted for 7.7%. These findings underscore the importance of systematic cardiac and renal evaluation in patients with situs inversus, even in the absence of overt clinical symptoms [6]. 

## Conclusions

This case highlights the significance of early identification of situs inversus in neonates when presenting with respiratory distress. While Kartagener's syndrome is a common condition associated with situs inversus, not every patient will exhibit the complete triad or have recognizable genetic variants associated with primary ciliary dyskinesia. In this case, genetic testing identified likely pathogenic variants in LAMA2 and NDUFAF5, neither of which is associated with laterality defects, suggesting the patient's situs inversus is likely idiopathic.

Documenting cases like this is important to increase awareness of situs inversus presentation, unrelated to Kartagener's syndrome. Additionally, it highlights the importance of comprehensive genetic testing to rule out known causes of isolated situs inversus, identify incidental yet potentially significant findings, and inform ongoing monitoring for potential neuromuscular or metabolic disorders. Collectively, documenting and studying similar cases may help us determine the genetic basis of isolated situs inversus and improve diagnostics and management in the future.
